# Distinct brain structural abnormalities in attention-deficit/hyperactivity disorder and substance use disorders: A comparative meta-analysis

**DOI:** 10.1038/s41398-022-02130-6

**Published:** 2022-09-06

**Authors:** Yajing Long, Nanfang Pan, Shiyu Ji, Kun Qin, Ying Chen, Xun Zhang, Min He, Xueling Suo, Yifan Yu, Song Wang, Qiyong Gong

**Affiliations:** 1 Huaxi MR Research Center (HMRRC), Department of Radiology, West China Hospital of Sichuan University, Chengdu, China; 2 Research Unit of Psychoradiology, Chinese Academy of Medical Sciences, Chengdu, China; 3 Functional & Molecular Imaging Key Laboratory of Sichuan Province, West China Hospital of Sichuan University, Chengdu, China; 4 Department of Biochemistry and Molecular Biology, Bloomberg School of Public Health, Johns Hopkins University, Baltimore, MD USA; 5 Department of Psychiatry, University of Cincinnati, Cincinnati, OH USA; 6 Department of Radiology, West China Xiamen Hospital of Sichuan University, Xiamen, China

**Keywords:** ADHD, Addiction

## Abstract

As two common mental disorders during the period of adolescence that extend to early adulthood, attention-deficit/hyperactivity disorder (ADHD) and substance use disorders (SUDs) have considerable diagnostic co-occurrence and shared neuropsychological impairments. Our study aimed to identify overlapping and distinct brain structural abnormalities associated with ADHD and SUDs among adolescents and young adults. A systematic literature search on voxel-based morphometry (VBM) studies of ADHD and SUDs was conducted in PubMed and Web of Science. Data were extracted and analyzed to identify brain abnormalities using Seed-based d-Mapping software. Data-driven functional decoding was conducted to identify the psychophysiological functioning associated with brain alterations. 13 and 14 VBM studies for ADHD (619 patients and 483 controls) and SUDs (516 patients and 413 controls), respectively, were included. Patterns of decreased gray matter volume (GMV) were found in the left precentral gyrus, bilateral superior frontal gyri, and left inferior frontal gyrus in the ADHD group compared to the control group. In contrast, individuals with SUDs, relative to controls, were characterized by increased GMV in the left putamen and insula. Comparative analysis indicated larger regional GMV in the right inferior parietal lobule and smaller volumes in the left putamen and left precentral gyrus in the ADHD group than in the SUDs group. Dissociable brain structural abnormalities in adolescents and young adults with ADHD and SUDs potentially implicate different pathogeneses and provide a reference for differential diagnosis and early detection for shared symptomology and comorbidity.

## Introduction

Attention-deficit/hyperactivity disorder (ADHD) and substance use disorders (SUDs) are distinct psychiatric conditions in the current categorical and hierarchical diagnostic system [1]. ADHD is a common neurodevelopmental disorder beginning in childhood and persisting into adolescence and even adulthood, depicted by inattention, hyperactivity, and impulsivity [1, 2]. SUDs present clusters of cognitive and behavioral symptoms caused by pathological patterns of substance use encompassing 10 separate classes of substances, including alcohol, cannabis, hallucinogens, and others [1]. More than 1/3 of individuals with ADHD were diagnosed with SUDs, and the prevalence of ADHD among adolescents and young adults with SUDs was reported to be up to 25.3% and 21.0%, respectively, indicating high co-occurrence in these two populations [3, 4]. In addition, children and adolescents with ADHD were more than 1.5 times as likely as healthy individuals to develop SUDs [5], suggesting that early ADHD is a risk factor for SUDs [6]. Co-occurrence of ADHD and SUDs have been shown to culminate in worse clinical manifestations and poorer prognosis, bringing about heavy public health burdens [7, 8].

Impulsivity caused by deficits in inhibitory control and reward processing is the most striking behavioral trait common to ADHD and SUDs [9, 10]. On the one hand, impulsivity is described as the failure of behavioral inhibition triggered by dysfunction of top-down executive control mediated by the prefrontal-parieto-striatal network [1, 11, 12]. On the other hand, atypical reward processing accounts for impulsive decision-making, manifested as greater delay discounting [13, 14] and high risk taking [15, 16]. Previous research has elucidated the cortico-basal ganglia circuits centered on the ventral striatum as the reward processing network [17]. The high comorbidity and overlapping behavioral profiles suggest potential shared neural substrates across disorders, indicating transdiagnostic neural biomarkers. In this regard, structural magnetic resonance imaging studies using voxel-based morphometry (VBM) approaches may provide empirical support.

Neuroimaging studies on ADHD have identified the delayed maturation of brain structure and function, particularly the prefrontal cortex and subcortical regions engaged in cognitive, attentional, and emotional processes [18–20]. Specifically, individuals with ADHD show consistent patterns of reduced gray matter volume (GMV) in the frontal-striatal circuitry comprised of the orbitofrontal cortex, anterior cingulate cortex (ACC), and striatum [21–24]. Hypoactivation of this circuitry was observed during inhibition tasks in individuals with ADHD relative to controls [25, 26], validating its regulatory role in abnormal inhibitory function [27]. In addition, individuals with ADHD also manifest brain abnormalities in reward-related structures and activation patterns [28]. The ventral striatum, the most prominent component of the reward system, exhibits smaller volume [19, 29] as well as lower activation during reward anticipation in those with ADHD [30]. Furthermore, ADHD subjects at high risk for developing SUDs showed increased activation in the reward processing network during impulsivity-related tasks, suggesting a hyperactive reward system as the potential cause underlying this comorbidity [31].

Similarly, individuals with SUDs present neuroadaptations in the frontal-striatal circuitry with reduced GMV in the prefrontal cortex, ACC, bilateral insula, and thalamus [32–34]. Impairments in reward processing have been evidenced by functional abnormalities in the striatum involved in habit formation, compulsive behavior, and reinforcement learning [35]. Subjects with SUDs showed striatal hypoactivation during reward anticipation compared with healthy controls, indicating reduced striatal responses to nondrug rewards [36]. In addition, functional alterations in the prefrontal regions during cognitive task performance mediate the dysfunction in executive and behavioral control contributing to the development of SUDs [35]. Remarkably, although individuals with SUDs and those with ADHD both exhibited inhibition-related brain abnormalities compared with healthy controls, different patterns of neural activation and recruited networks were involved [37].

Identifying their common neural phenotypes may help to detect those who have high vulnerability to comorbidity, which would allow early intervention, and exploring disjunctive neural properties may facilitate differential diagnosis. Nevertheless, due to the lack of research directly comparing brain structural alterations between those with ADHD and SUDs, it remains unclear whether there are common or disorder-specific structural brain abnormalities. Therefore, we conducted a voxel-based neuroimaging meta-analysis to explore the overlapping and distinct brain regional volumetric changes between individuals with ADHD and SUDs. Given the high prevalence of co-occurrence during the period of adolescence and early adulthood, we selected adolescents and young adults for this analysis and defined this population as 12–24 years old [38]. To provide an objective and quantitative interpretation of our findings, we ascertained the psychological functions of the identified clusters via data-driven functional decoding. By identifying overlapping and distinguishable neuroanatomical abnormalities, we hope to provide insights into the underlying neuropathological mechanisms that have implications in clinical settings.

## Methods

### Literature search and study selection

The study protocol was preregistered on the Open Science Framework (https://osf.io/r5xz2). Since the first method paper about VBM was published in 2000 [39], the retrieval date was set from January 1999 to March 2021. The literature search was conducted systematically and comprehensively by two authors (Y. J. and J. S.) from the databases of PubMed and Web of Science based on the Preferred Reporting Items for Systematic Reviews and Meta-Analyses (PRISMA) guidelines [40]. Additionally, manual searches were conducted among reference lists of previous VBM meta-analysis studies (details of the search in supplementary methods). The inclusion criteria were as follows: 1) original study compared individuals with ADHD or SUDs against healthy controls on regional GMV; 2) VBM method was utilized; 3) whole-brain gray matter results with peak coordinates of the brain regions were reported (including non-significant results) rather than only region of interest (ROI) outcomes. Studies were excluded if they: 1) reported duplicate data from other publications (including meta-analysis and mega-analysis); 2) included participates aged <12 years or >24 years; 3) involved less than 10 subjects per group.

### Data selection and extraction

The screening and assessing processes for each article were independently performed by two authors (Y. L. and S. J.). If two researchers had inconsistent opinions on the inclusion or exclusion of one study, they would discuss with the third author (N. P.) to reach a consensus. We recorded sample size, mean age, number of female subjects, comorbidity and medication status, scanner and preprocessing protocols, statistical approach as well as peak coordinates for brain structural abnormalities and corresponding statistical values of each study to construct the database.

### Meta-analysis for VBM studies

Prior to meta-analyses, age and sex were compared across patient and control groups in SPSS Statistics, version 24. Separate meta-analysis of regional differences of brain gray matter in populations with ADHD and SUDs was conducted using Anisotropic Effect Size Seed-based *d*-Mapping (AES-SDM) software (https://www.sdmproject.com/old/) respectively. AES-SDM is a statistical technique that uses the altered cluster information reported in individual studies to recreate the statistical effect-size maps when considering their variances with the approach of anisotropic Gaussian kernel [41, 42]. Text files were obtained from included studies containing information about peak coordinates and corresponding statistical values. A map of *d* values and a map of their variances were created and combined to obtain the meta-analytic maps in the preprocessing. Then statistical maps were generated in the main analysis utilizing standard random-effects general linear model, with a *p* < 0.001 threshold for this step [42, 43]. We set the peak height threshold as 1.000 and only the cluster with more than 10 voxels would be counted [42, 44]. Notably, the cluster size of identified cluster represents the explanatory weight of the clinical question explored and larger clusters in our study correspond to more significant neural abnormalities in ADHD or SUDs group.

Following the separate disorder-specific analysis, we further conducted the overlapping and comparative analyses by applying the multimodal and linear models to assess whether there were any common or distinct structural alterations across two groups by comparing ADHD and SUDs groups directly (threshold *p* < 0.001 and cluster size > 10 voxels) [42]. To account for the heterogeneity between studies, meta-regression analysis was performed to examine the potential effect of demographic factors at the whole-brain level [42]. Subsequently, funnel plots and Egger’s tests for potential publication bias were examined additionally [45, 46]. Jack-knife sensitivity analyses were conducted to assess the replicability and robustness of the findings by repeating the mean statistical analysis discarding one study out at a time [42].

### Functional decoding of identified clusters

To explore the psychological process relevant to each identified brain region, we performed a functional decoding analysis by retrieving related psychophysiological terms to brain alterations in NeuroSynth decoder (https://neurosynth.org/decode/) [47, 48]. Brain statistical maps were uploaded to and analyzed by the NeuroSynth which combined text mining, mega-analysis, and machine learning approaches to obtain probabilistic mappings between psychological topics and neural states [47]. We classified those psychological terms and then calculated the correlation coefficients by averaging values corresponding to behavioral domains based on the taxonomy on BrainMap (https://www.brainmap.org/taxonomy/behaviors.html) [49, 50]. All psychological experiments can be categorized into 5 main behavioral domains (cognition, action/motor, emotion, perception, interoception) as well as their subcategories in terms of neural/behavioral systems studied [49]. Therefore, we centered on these 5 behavioral domains to identify the most prominent behavioral domain associated with suprathreshold brain regions [50].

## Results

### Search results and sample characteristics

**Fig. 1:**
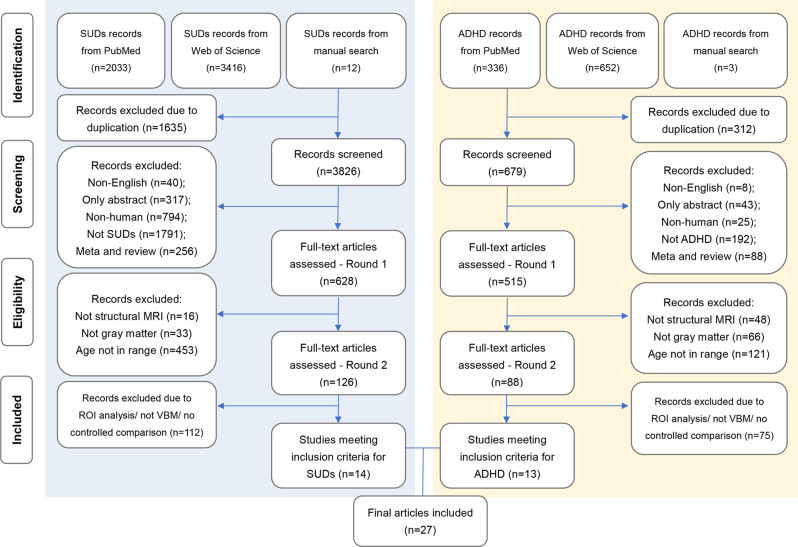
Flowcharts of the literature search and selection criteria for articles on attention deficit/hyperactivity disorder (ADHD) and substance use disorders (SUDs) in the meta-analysis. MRI Magnetic resonance imaging, ROI Region of interest, VBM Voxel-based morphometry.

In total, 13 studies for ADHD and 14 studies for SUDs were included after a systematic literature search (procedures of literature search in Fig. 1) incorporating observations from 619 ADHD subjects (mean age 15.43, 23.15% female) and 516 SUDs subjects (mean age 19.76, 35.09% female) as well as 896 healthy controls. Individuals with SUDs were older (*p* < 0.001) and consisted of a larger proportion of females (*p* < 0.001) than those with ADHD. In the SUDs group, a total of 8 substances of interest were investigated, including stimulants (31.98%), cannabis (23.84%), alcohol (17.05%), tobacco (15.12%), inhalants (2.91%), and poly-substance use (9.11%). Demographic characteristics and other details of included sample in Supplementary Tables S1, S2.

### Meta-analysis of regional gray matter alterations

#### ADHD versus healthy controls

**Fig. 2:**
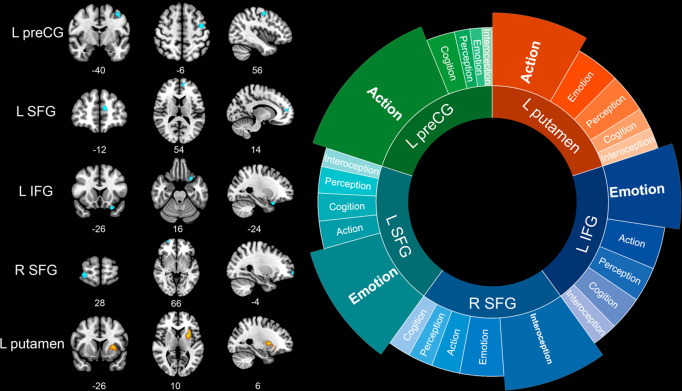
Brain abnormalities of attention deficit/hyperactivity disorder (ADHD) and substance use disorders (SUDs) relative to controls and their corresponding functioning. Clusters were exhibited in the sagittal, axial, and coronal planes at *p* < 0.001, *z* > 1, and cluster size > 10 voxels. Increased GMV patterns (SUDs) were shown in orange while decreased patterns (ADHD) in blue. Results of functional decoding presented contribution of each behavioral domain to each suprathreshold cluster. L Left, R Right, preCG Precentral gyrus, SFG Superior frontal gyrus, IFG Inferior frontal gyrus.

Reduced GMV patterns were found in the ADHD group, including the left precentral gyrus (preCG, *x* = −40, *y* = −6, *z* = 56; *Z* = −2.924; cluster size = 97), bilateral superior frontal gyri (SFG, left: *x* = −12, *y* = 54, *z* = 14; *Z* = −3.194; cluster size = 73; right: *x* = 28, *y* = 66, *z* = −4; *Z* = −2.882; cluster size = 38), orbital part of the left inferior frontal gyrus (IFG, *x* = −26, *y* = 16, *z* = −24; *Z* = −3.003; cluster size = 52) (Table 1 and Fig. 2). As presented in Fig. 2, functional decoding exhibited that the left preCG was predominantly associated with the action domain, while both left SFG and IFG were closely associated with the emotion domain. In contrast to the left SFG, the right SFG was mostly related to the interoception domain.

**Table 1:** Regional differences of gray matter volume in and across attention-deficit/hyperactivity disorder (ADHD)/substance use disorders (SUDs) group^a^.

Contrast/Brain region	MNI coordinate	SDM-Z	*P*-value	Cluster size	BA
**ADHD vs. HC**					
**ADHD** **<** **HC**					
L precentral gyrus	–40, –6, 56	−2.924	0.0002	94	6
L superior frontal gyrus	−12, 54, 14	−3.194	<0.0001	69	10/32
L inferior frontal gyrus	−26, 16, −24	−3.003	<0.0001	50	38
R superior frontal gyrus	28, 66, −4	−2.882	0.0002	35	11
**SUDs vs. HC**					
**SUDs** **>** **HC**					
L putamen	−26, 10, 6	1.926	<0.0001	530	48/47
**ADHD vs. SUDs**					
**ADHD** **>** **SUDs**					
R inferior parietal lobule	54, −28, 52	1.779	0.0005	12	1/2
**ADHD** **<** **SUDs**					
L putamen/insula	−28, 22, 0	−2.028	0.0003	271	47/48
L precentral gyrus	−40, −6, 54	−2.066	0.0002	187	6

^a^Significant clusters were identified at *p* < 0.001 and cluster size > 10 voxels.

*BA* Brodmann area, *L* Left, *R* Right, *MNI* Montreal Neurological Institute.

#### SUDs versus healthy controls

SUDs group had greater volumetric alterations in the left putamen (extending to insula) (*x* = −26, *y* = 10, *z* = 6; *Z* = 1.926; cluster size = 530) compared with control group (Table 1 and Fig. 2). Functional decoding results revealed that the most pertinent behavioral domain was action (Fig. 2).

#### Overlapping and comparative analysis between disorders

**Fig. 3:**
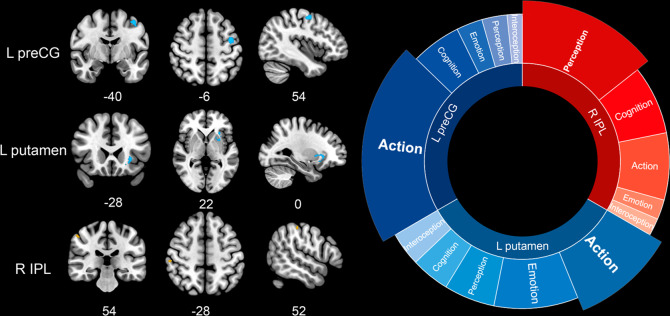
Comparative brain abnormalities of attention deficit/hyperactivity disorder (ADHD) to substance use disorders (SUDs) and their corresponding functioning. Clusters were exhibited in the sagittal, axial, and coronal planes at *p* < 0.001, *z* > 1, and cluster size > 10 voxels. Increased GMV patterns of ADHD relative to SUDs were shown in orange while decreased patterns in blue. Results of functional decoding presented contribution of each behavioral domain to each suprathreshold cluster. L Left, R Right, preCG Precentral gyrus, IPL Inferior parietal lobule.

The overlapping analysis did not yield any significant results. However, the comparative analysis found that individuals with ADHD had consistently disorder-differentiating increased GMV in the right inferior parietal lobule (IPL, *x* = 54, *y* = −28, *z* = 52; *Z* = 1.779; cluster size = 12), and reduced GMV in the left putamen (extending to left insula) (*x* = −28, *y* = 22, *z* = 0; *Z* = −2.028; cluster size = 70) and left preCG (*x* = −40, *y* = −6, *z* = 54; *Z* = −2.066; cluster size = 72) relative to those with SUDs (Table 1 and Fig. 3). The right IPL, in accordance with its anatomical functions, had tight bonds with perception domain (Fig. 3). Clusters in the left putamen and left preCG identified in the exploratory linear model were located similarly as those found in meta-analysis of ADHD. Results of functional decoding were similar as well that the putamen was associated with action and emotion domain, while the left preCG with action domain (Fig. 3).

#### Meta-regression analysis

**Fig. 4:**
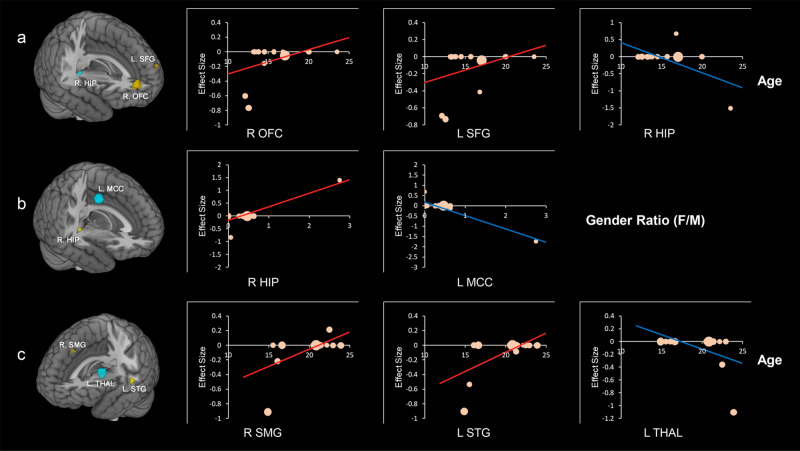
Results of meta-regression analyses. **a** Brain regions where the associations of attention deficit/hyperactivity disorder (ADHD) with GMV were modulated by age. **b** Brain regions where the associations of ADHD with GMV were modulated by sex (female ratio). **c** Brain regions where the associations of substance use disorders with GMV were modulated by age. Clusters were displayed at *p* < 0.0005 and cluster size > 10 voxels. Positive correlation was shown in orange with an upward regression line while negative patterns in blue with a downward line. In the plot, each study is marked as a dot, and the size of each dot corresponds to the sample size. L Left, R Right, SFG Superior frontal gyrus, HIP Hippocampus, OFC Orbitofrontal cortex, MCC Median cingulate gyrus, SMG Supramarginal gyrus, STG Superior temporal gyrus, THAL Thalamus, F/M Female/male.

For ADHD, meta-regression analyses revealed that larger volumes relative to controls were associated with increasing age in the bilateral SFG. On the contrary, smaller volume was associated with greater age compared with controls in the right hippocampus. Additionally, studies with a higher proportion of female found larger GMV compared with controls in right hippocampus, while left middle cingulate gyrus volume was negatively correlated with the proportion of female. Regarding SUDs, larger volumes compared with controls were associated with higher age in the thalamus, right supramarginal gyrus, and the left superior temporal gyrus (Fig. 4 and Table S3). Our study did not recognize any effect of sex on regional GMV in SUDs.

#### Publication bias and jack-knife sensitivity findings

For ADHD, Egger’s tests revealed publication bias in several clusters encompassing the left preCG (*p* < 0.001), left IFG (*p* < 0.001), and the right SFG (*p* < 0.001), yet funnel plots were found to be symmetric across all clusters. Publication bias was not significant with respect to GMV differences found in left putamen in SUDs studies. Jack-knife analyses confirmed the robustness of our main findings without apparent fluctuation, in which identified clusters could be replicated in 12 out of 13 primary ADHD studies and 13 out of 14 SUDs studies (Table S4).

## Discussion

To the best of our knowledge, this is the first whole-brain neuroimaging meta-analysis that aimed to disentangle the neural structural correlates and differences associated with ADHD and SUDs among adolescents and young adults. The initial meta-analysis for each disorder found that ADHD and SUDs had substantial disorder-specific GMV alteration patterns relative to healthy controls, with decreased GMV in the left preCG, bilateral SFG, and left IFG in those with ADHD but increased GMV in the left putamen in those with SUDs. Comparative analysis revealed that individuals with ADHD presented larger GMV in the IPL and smaller GMV in the putamen and preCG than those with SUDs. Functional decoding indicated that these abnormalities mainly corresponded to perception and action. Overall, our findings showed that altered patterns of brain gray matter structure associated with ADHD and SUDs are spatially discordant during the period of puberty and young adulthood, which may facilitate differential diagnosis in clinical settings.

### ADHD-related GMV alterations

In the disorder-specific meta-analysis, we found that decreased GMV in the left preCG differentiated ADHD. The preCG is a key region engaged in fine motor control and direct sensorimotor mappings [51] and is one of the neuropathological markers of individuals with ADHD [52, 53]. Decreased activation in the preCG has been associated with poor executive functions observed in individuals with ADHD, manifesting dysfunction in response inhibition, sustained attention, and task switching [54, 55]. In addition, the somatosensory network, including the preCG, showed hypoconnectivity in those with ADHD, which may account for motor hyperactivity and impulsivity symptoms [56].

Patterns of GMV reduction were also found in the bilateral SFG and orbital part of the IFG, showing good convergence across prior studies [57, 58]. These frontal regions correspond to superior cognitive control and emotion regulation [59–61], and dysfunction in these regions contributes to substantial deficits in executive and affective control in ADHD [55, 62]. In addition, the orbital part of the IFG is located at the overlapping area of the IFG and the orbitofrontal cortex, showing rich interconnections with the amygdala, thalamus, and other subcortical regions [63]. This region has frequently been identified as a transdiagnostic key node in multiple cognitive control and emotion evaluation-related neural circuits across a wide range of psychiatric disorders in young populations, especially those with ADHD [64]. The GMV reduction in the orbital part of the IFG supported the notion that emotion dysregulation in ADHD may be triggered by defective processing of emotional cues and an inability to maintain emotional homeostasis [65, 66]. Additionally, these frontal loci were highlighted as pivotal components in the default mode network (DMN) [67], whose enhanced activation and disrupted connectivity were of clinical relevance with inattention in ADHD [68, 69].

### SUDs-related GMV alterations

Increased GMV in the left putamen extending to the insula in the SUDs group was identified. As part of the frontal-striatal circuitry, the putamen receives glutamatergic and dopaminergic inputs and coordinates various aspects of motion and cognition, serving as a crucial component of the motor and reward systems in addictive behaviors [70, 71, 72]. Structural and functional abnormalities in the putamen have been associated with elevated relapse vulnerability and the transition from voluntary to compulsive drug use driven by craving and habit learning among drug-dependent subjects [73–76].

Notably, partial enlargement of the insula was also observed in the SUDs group compared with the control group. From a psychological perspective, the insula is recognized as an integrator between emotional, cognitive, and sensory-motor systems [77]. Analogous to the putamen, dysfunction in the insula prompted craving, drug-seeking behaviors, and relapse by strengthening interoceptive processing related to substance use [78, 79]. Several studies have confirmed that individuals with SUDs with lesions to the insula and the adjacent putamen could abstain from smoking more easily without undergoing craving or relapse [80, 81].

### Differentiating GMV alterations between ADHD and SUDs

Comparative patterns were detected in the right IPL, where the ADHD group showed an enlarged GMV compared to the SUDs group, and in the left putamen and left preCG, where the reverse pattern was observed. Given the GMV reduction in the preCG in those with ADHD and the increased GMV in the putamen in those with SUDs were reported when compared with controls, differences in the above two clusters naturally met our expectation. Thus, the right IPL stood out as a region that distinguished the disorders. From the perspective of local neuropsychological functions, the IPL mediates the superior processing of motor and sensory information and attention [82]. When considering its role in large-scale networks, the IPL forms the executive control network together with prefrontal areas, which is involved in the regulation of inhibitory control [83]. In those with ADHD, hypoactivation in the IPL was detected during various cognitive tasks, partially accounting for cognitive deficits [84–86]. Impaired activation of the IPL has also been associated with inattention and impulsivity symptoms of ADHD given its regulatory and guiding effect on attention processing [85, 87]. In those with SUDs, structural alterations in the IPL have rarely been reported. However, the reduced GMV in the parietal cortex and abnormal neural activation patterns in the frontoparietal network (hypoactivation during working memory and hyperactivation during response inhibition) might predict the development of SUDs in adolescents and young adults [88].

We initially speculated that the alterations in inhibitory control and reward processing-related neural structures might be the overlapping mechanisms underlying ADHD and SUDs. However, discrepant structural abnormalities in the early life stages of the two disorders were found, indicating distinct differentiating neural signatures. We inferred from the results that, although two disorders ended up affecting similar circuits, abnormalities in ADHD may initially originate from immature frontal cortices and progress into regions governing motor control and the reward system, which is a form of top-down regulation [89]. In contrast, the neuropathological processing underlying SUDs could be triggered by drug-related adaptations in neural systems, mainly a hyperactive reward system, which initially occurs in the striatum [9, 90]. Impelled by up-regulated motivation, cravings, and reinforcing effects of drugs, damage with continuous substance administration extends to prefrontal circuits, leading to impaired executive function [91–93]. Such an inference sheds light on the understanding of the underlying pathophysiology and may aid in clinical settings. Distinguishable brain structural patterns enable the early screening and differential diagnosis of ADHD and SUDs.

### Potential effects of age and sex

The meta-regression analyses showed sources of the heterogeneity among demographic variables associated with brain abnormalities contributing to these disorders of interest. Age exhibited a modulatory effect on the regional GMV alterations in the bilateral SFG in individuals with ADHD. Children with ADHD presented with decreased GMV in frontal areas triggered by a developmental delay of neural maturation [18]. The structural discrepancies in the frontal cortex between ADHD subjects and typically developing populations would gradually diminish from childhood into adulthood [18, 94], along with the remission of symptoms based on longitudinal cohorts. The SFG has been related to cognitive dysfunction in the neuropathological development of ADHD [94, 95]. Modulatory effects in the SFG may account for the decreased severity of executive dysfunction with increasing age of individuals with ADHD [96, 97]. We also found that hippocampal GMV was positively correlated with the proportion of females in the ADHD group. Notably, previous studies found that boys with ADHD displayed a volumetric reduction in subcortical regions that girls did not show [98, 99]. Our study further confirmed the inverse patterns of sex in volumetric hippocampal abnormalities in ADHD. Regarding SUDs, we observed modulatory effects of age on GMV alterations in the thalamus. This finding indicated that the trajectories of thalamic volume alterations in individuals with SUDs differed from those in typically developing individuals with age-related atrophy [100, 101].

### Limitations and future perspectives

Our study had several limitations for further consideration. First, given that all the included studies were cross-sectional, causal interpretations of these findings may not be sensible [102]. It is suggested that future studies employ a longitudinal design and recruit matched groups of individuals with the two disorders to directly compare the abnormalities in brain GMV. Second, due to the limited number of included articles focusing on a specific type of substance addiction, performing subgroup analyses could not be conducted. The neuroadaptations in those with SUDs vary in anatomical morphology when taking the types of addictive substances into account [32]. In addition, we failed to conduct a subgroup analysis to exclusively examine the effects of comorbidity due to the limited number of articles with detailed descriptions of comorbid conditions. Third, the diversity of preprocessing protocols (analytical software, smoothing kernel size and statistical thresholds) among the included studies might have produced considerable heterogeneity [103]. We conducted jack-knife sensitivity analyses to assess the robustness of our findings, and the results were reproducible.

## Conclusion

Although ADHD and SUDs share neuropsychological features and a high level of co-occurrence among adolescents and young adults, they exhibited distinct patterns of GMV alterations. Decreased GMV was observed in the motor cortex and frontal lobes in ADHD patients compared with healthy controls, while an increased volumetric pattern in the left putamen was observed in those with SUDs. The ADHD group showed larger regional GMV in the right IPL and smaller volumes in the left putamen and left preCG than the SUDs group. These patterns of alterations may correspond to various types of psychopathological processing in the action and perception domains in two disorders of interest. From an objective view, the current findings elucidate distinct brain structural abnormalities between ADHD and SUDs, which may pave the way for a better understanding of the differentiation in clinical settings. In addition, our study may contribute to the development of psychoradiology [104], which is an emerging field on the application of imaging techniques to psychiatric conditions [105–110].

## Supplementary information

Supplemental Material
