# Supplementary material for: Distinct brain structural abnormalities in attention-deficit/hyperactivity disorder and substance use disorders: A comparative meta-analysis
Source: Transl Psychiatry. 2022 Sep 6;12:368. doi: 10.1038/s41398-022-02130-6 (PMC9448791; doi:10.1038/s41398-022-02130-6)
Supplement: Supplementary file 1 — Supplemental Material [file 41398_2022_2130_MOESM1_ESM.docx]

**Distinct Brain Structural Abnormalities in Attention-Deficit/Hyperactivity Disorder and Substance Use Disorders: A Comparative Meta-analysis**

**Supplementary Methods**

Appendix 1. Literature search

**Supplementary Tables and Figures**

Table S1. Demographic characteristics of included sample

Table S2. Details of the studies included in the meta-analysis

Table S3. The results of the meta-regression analyses showing effects of age and gender on disorder-gray matter volume (GMV) relationship

Table S4. Jack-knife sensitivity analyses of the studies included in the meta-analysis

**References**

**Appendix 1. Literature search**

We searched for studies that investigate the brain abnormalities of adolescents and young adults with attention-deficit/hyperactivity disorder (ADHD) or substance use disorders (SUDs) on voxel-based morphometry (VBM) of structural magnetic resonance imaging (sMRI). According to pre-registered protocol (<https://osf.io/r5xz2>), we conducted the literature search in PubMed ((<https://pubmed.ncbi.nlm.nih.gov/>) and Web of Science (<https://apps.webofknowledge.com>) with the retrieval date from January 1999 to March 2021. Search strategy utilized for substance misuse studies was ("substance-related disorders" OR "substance use disorder" OR "SUD" OR "substance abuse" OR "drug abuse" OR "addiction" OR "alcohol" OR "AUD" OR "alcohol use disorder" OR “caffeine” OR "marijuana" OR "cannabis" OR “hallucinogens” OR "inhalant" OR “opioid” OR “sedatives” OR “hypnotics” OR “anxiolytics” OR "amphetamine" OR "methamphetamine" OR "MDMA" OR "cocaine" OR "nicotine" OR "smoking" OR "smoker" OR “tobacco” OR “cigarette” OR "ecstasy" OR "heroin" OR “benzodiazepine” OR “psychoactive substances”) AND ("Morphometry" OR "Voxel based" OR "VBM" OR "voxel wise" OR “gray matter” OR “grey matter”) and the search strategy for ADHD studies was ("ADHD" OR "Attention deficit hyperactivity disorder") AND ("Morphometry" OR "Voxel based" OR "VBM" OR "voxel wise" OR “gray matter” OR “grey matter”). We noticed that meta-analyses separately exploring the neural correlates of ADHD and SUDs already exist [1-3]. However, to ensure the consistency of inclusion and exclusion criteria and to avoid possible omissions, we still performed a comprehensive literature search and manually add records tracing of previous meta-analysis studies.

**Table S1. Demographic characteristics of included sample**

|  | **ADHD** | **SUDs** | ***t* value ^a^** | **HC_ADHD_** | **HC_SUDs_** |
| --- | --- | --- | --- | --- | --- |
| N | 619 | 516 | - | 483 | 413 |
| Mean age, y | 15.43 | 19.76 | -23.657 ^b^ | 15.48 | 19.86 |
| % Females | 23.15% | 35.09% | -9.840 ^b^ | 31.06% | 40.19% |

^a^ Independent sample t-test.

^b^ *p* < 0.05.

Abbreviations: ADHD, attention-deficit/hyperactivity disorder; HC, healthy control; SUDs: substance use disorders; % Females, for the proportion of females in whole sample.

**Table S2. Details of the studies included in the meta-analysis**

| **Attention-Deficit/Hyperactivity Disorder** | | | | | | | | | | | |
| --- | --- | --- | --- | --- | --- | --- | --- | --- | --- | --- | --- |
| **Study** | **Group** | **N(Male)** | **Mean age (SD)** | **Subtype** | **Current axis I comorbidity** | **Proportion of medication (in past 2 weeks)** | **Main findings** | **Scanner /FWHM (mm)** | **Slice thickness (mm)** | **Preprocessing Software** | **Statistical level** |
| Bonath et al. 2018 | ADHD | 18(18) | 13.6(1.7) | C/I | ODD (1) | 0.56 | ADHD<HC: Cerebellum, Occipital gyrus, ACC, HPC, AMG | 3/8 | 1 | SPM8 | p<0.05, FDR |
|  | HC | 18(18) | 14.1(1.3) |  |  |  |  |  |  |  |  |
| Bralten et al. 2016 | ADHD | 307(209) | 17.06(3.42) | C/I | - | 0.89 | ADHD<HC: PreCG, OFC, frontal gyrus, CG | 1.5/8 | 1 | SPM8 | p<0.05, FWE |
|  | HC | 196(96) | 16.66(3.07) |  |  |  |  |  |  |  |  |
| Brieber et al. 2007 | ADHD | 15(15) | 13.13(1.4) | C/I | - | 0.67 | ADHD<HC: CN, HPC, MFG, INS, STG, MOG | 1.5/12 | 1 | SPM2 | p<0.001, uncorrected |
|  | HC | 15(15) | 13.3(1.8) |  |  |  | ADHD>HC: SPL, poCG, MCC, PCUN, IPL |  |  |  |  |
| Depue et al. 2010 | ADHD | 31(19) | 20.0(1.7) | C | - | 0.77 | NS | N/A/4.6 | 1.7 | FSL | Monte Carlo |
|  | HC | 21(8) | 19.3(1.1) |  |  |  |  |  |  |  |  |
| Iannaccone et al. 2015 | ADHD | 18(11) | 14.50(1.52) | C | affective disorder (2); adjustment disorder (3); anxiety disorder/phobias (3); dyscalculia (2); CD (2) | 0.72 | ADHD<HC: ACC, SFG, SMA, CG, cerebellum | 3/9 | 1 | SPM8 | Monte Carlo |
|  | HC | 18(9) | 14.82(1.24) |  |  |  | ADHD>HC: poCG, PreCG |  |  |  |  |
| Kappel et al. 2015 | ADHD | 16(15) | 23.5(4.1) | C | - | 0.00 | ADHD<HC: SMG, PCUN, HPC, OFC, rectal gyrus | 3/6 | 1 | SPM8 | p<0.05, AlphaSim |
|  | HC | 20(20) | 23.7(3.4) |  |  |  |  |  |  |  |  |
| Lim et al. 2013 | ADHD | 29(29) | 13.8(1.84) | C | - | 0.21 | ADHD<HC: Cerebellum, IFG, IPL, temporal gyrus | 3/8 | N/A | SPM8 | p<0.001, uncorrected |
|  | HC | 29(29) | 14.4(2.48) |  |  |  |  |  |  |  |  |
| Ramesh et al. 2013 | ADHD | 15(4) | 16.8(2.5) | C | N/A | N/A | ADHD<HC: Occipital gyrus, CG, SFG | N/A/8 | 1 | SPM8 | p<0.05, FDR |
|  | HC | 15(4) | 16.72(2.55) |  |  |  | ADHD>HC: Thalamus |  |  |  |  |
| Saad et al. 2017 | ADHD | 34(25) | 13.28(2.75) | C/I | ODD (10) | 0.38 | NS | 3/N/A | 1 | SPM8 | p<0.05, FWE |
|  | HC | 28(19) | 13.09(2.63) |  |  |  |  |  |  |  |  |
| Stevens et al. 2012 | ADHD | 24(19) | 15.7(1.55) | C | - | N/A | ADHD<HC: preCG, IFG | 3/8 | 1 | SPM8 | p<0.05, FDR |
|  | HC | 24(16) | 16.0(1.47) |  |  |  | ADHD>HC: caudate, VLPFC |  |  |  |  |
| Van Dessel et al. 2020 | ADHD | 28(28) | 14.5(2.1) | C | N/A | N/A | ADHD<HC: AMG, MeTG | 3/8 | 1 | SPM12 | p<0.05, FWE |
|  | HC | 32(32) | 14.7(2.1) |  |  |  |  |  |  |  |  |
| Vilgis et al. 2016 | ADHD | 48(48) | 12.58(2.21) | C | anxiety disorders (14); persistent depressive disorder (8); ODD (18); CD(1) | 0.25 | ADHD<HC: frontal gyrus, SPL, PCUN, MTG | 3/8 | 1 | SPM12 | p<0.05, FWE |
|  | HC | 31(31) | 12.75(1.96) |  |  |  |  |  |  |  |  |
| Zhao et al. 2020 | ADHD | 36(36) | 12.14(2.12) | C | - | N/A | ADHD<HC: SFG, MFG, cuneus, MCC | 3/8 | 1.3; 1 | SPM12 | p<0.001, FDR |
|  | HC | 36(36) | 11.69(1.70) |  |  |  |  |  |  |  |  |
| **Substance Use Disorders** | | | | | | | | | | | |
| **Study** | **Group** | **N(Male)** | **Mean age (SD)** | **Substance** | **Current axis I comorbidity** | **Proportion of medication (in past 2 weeks)** | **Main findings** |  |  |  | **Statistical level** |
| Aydin et al. 2009 | SUDs | 15 (15) | 15.53 (1.30) | Inhalants | - | 0.00 | SUDs<HC: MFG, OFC, SFG, AG, SPL, PHG, MTG | 1.5/10 | 1.3 | SPM5 | p<0.05, FWE |
|  | HC | 20 (20) | 15.60 (1.09) |  |  |  |  |  |  |  |  |
| Brooks et al. 2014 | SUDs | 58(25) | 14.9(0.8) | Alcohol | - | 0.00 | SUDs<HC: STG | 3/8 | 1 | SPM8 | p<0.05, FWE |
|  | HC | 58(25) | 14.7(0.8) |  |  |  |  |  |  |  |  |
| Bu et al. 2016 | SUDs | 26 (26) | 21.42 (1.73) | Tobacco | - | 0.00 | SUDs<HC: ACC | 3/8 | 1 | SPM8 | p<0.05, FWE |
|  | HC | 26 (26) | 20.58 (1.47) |  |  |  | SUDs>HC: Putamen |  |  |  |  |
| Cousijn et al. 2012 | SUDs | 33 (21) | 21.3 (2.4) | Cannabis | - | N/A | SUDs>HC: cerebellum | 3/8 | 1.2 | SPM8 | p<0.05, FWE |
|  | HC | 42 (26) | 21.9 (2.4) |  |  |  |  |  |  |  |  |
| M. Dalwani et al. 2011 | SUDs | 25 (25) | 16.64 (0.23) | Polysubstance (alcohol, cannabis, tobacco, club drugs, cacaine, hallucinogens, amphetamine ) | ADHD (3); CD (22) | N/A | SUDs<HC: DLPFC, IFG, LING, cerebellum | 3/8 | 1.7 | SPM5 | p<0.05, FWE |
|  | HC | 19 (19) | 16.59 (0.37) |  |  |  | SUDs>HC: Cuneus, PCUN |  |  |  |  |
| M. S. Dalwani et al. 2015 | SUDs | 22 (0) | 16.09 (0.20) | Polysubstance (alcohol, amphetamine, cannabis, club drugs, cacaine, hallucinogens, tobacco) | CD (14) | 0.41 | SUDs<HC: mPFC, VLPFC, DLPFC, mOFC, CG, somatosensory & motor cortex, SMG, AG | 3/8 | 1.7 | SPM8 | p<0.05, FWE |
|  | HC | 21 (0) | 16.67 (0.25) |  |  |  |  |  |  |  |  |
| Doallo et al. 2014 | SUDs | 11 (7) | 22.18 (1.08) | Alcohol | - | 0.00 | SUDs>HC: MCC, MOG, MFG, ACC, preCG | 1.5/6 | 1 | SPM8 | p<0.001, uncorrected |
|  | HC | 21 (10) | 22.43 (1.03) |  |  |  |  |  |  |  |  |
| Gilman et al. 2014 | SUDs | 20 (9) | 21.3 (1.9) | Cannabis | - | N/A | SUDs<HC: frontal pole, DLPFC, MFG, TP, STG, LOC | 3/6.9 | 1 | FSL | p<0.0005, uncorrected |
|  | HC | 20 (9) | 20.7 (1.9) |  |  |  | SUDs>HC: MFG, precuneus, PoCG, INS, putamen, AMG, occipital gyrus |  |  |  |  |
| Hanlon et al. 2016 | SUDs | 30 (21) | 23.9 (-) | Tobacco | - | 0.00 | SUDs<HC: AMG, thalamus | 3/8 | 1 | SPM8 | p<0.01, FWE |
|  | HC | 29 (14) | 23.0 (-) |  |  |  |  |  |  |  |  |
| Howell et al. 2013 | SUDs | 19 (7) | 22.95 (3.41) | Alcohol | - | 0.00 | SUDs<HC: PCUN | 3/10 | 1 | SPM8 | p<0.001, uncorrected |
|  | HC | 19 (7) | 24.63 (4.40) |  |  |  | SUDs>HC: ventral striatum, thalamus, LING |  |  |  |  |
| Koenders et al. 2016 | SUDs | 20 (15) | 20.5 (2.11) | Cannabis | alcohol use disorder (4); multiple substance abuse dependence (3); psychotic disorder (1); affective disorder (1); multiple psychiatric diagnoses (3) | N/A | NS | 3/8 | 1.2 | SPM8 | p<0.05, FWE |
|  | HC | 22 (14) | 21.6 (2.45) |  |  |  |  |  |  |  |  |
| Mackey et al 2014 | SUDs | 165(101) | 20.85 (1.52) | Stimulants | - | N/A | SUDs >HC: putamen | 3/7.06 | 1 | FSL | p<0.01, AlphaSim |
|  | HC | 46 (21) | 21.02 (2.17) |  |  |  | SUDs <HC: dorsolateral cerebellum, IPL |  |  |  |  |
| Wang et al. 2014 | SUDs | 22 (22) | 22.48 (2.48) | Tobacco | - | N/A | SUDs >HC: AG, IPL | 3/10 | 1 | SPM8 | p<0.05, AlphaSim |
|  | HC | 20 (20) | 21.80 (1.32) |  |  |  | SUDs<HC: INS, thalamus, MeFG |  |  |  |  |
| Weiland et al. 2015 | SUDs | 50 (41) | 16.65 (1.09) | Cannabis | - | 0.00 | NS | 3/6.9 | 1 | FSL | Monte Carlo |
|  | HC | 50 (36) | 16.77 (0.95) |  |  |  |  |  |  |  |  |

Abbreviations: N., number of subjects; subtype (C, combined; I, inattentive); statistical level (FDR, false discovery rate; FEW, family-wise error rate); SD, standard deviation; ADHD, attention-deficit/hyperactivity disorder; HC, healthy control; NS, not significant; N/A, not available; SUDs, substance use disorders; CD, conduct disorder; ODD, oppositional defiant disorder; SPM, Statistical Parametric Mapping; FSL, FMRIB Software Library.For the abbreviations of brain regions: ACC, anterior cingulate cortex; AG, angular gyrus; AMG, amygdala; CG, cingulate gyrus; CN, caudate nucleus; DLPFC, dorsolateral prefrontal cortex; HPC, hippocampus; IPL, inferior parietal lobule; INS, insula; IFG, inferior frontal gyrus; LING, lingual gyrus; LOC, lateral occipital cortex; MFG, middle frontal gyrus; MOG, middle occipital gyrus; MCC, middle cingulate cortex; MeFG, medial frontal gyrus; MeTG: medial temporal gyrus; MTG, middle temporal gyrus; mPFC, medial prefrontal cortex; mOFC, medial orbitofrontal cortex; OFC, orbitofrontal cortex; PCUN, precuneus; PHG, parahippocampal gyrus; preCG, precentral gyrus; poCG, postcentral gyrus; SFG, superior frontal gyrus; SMA, supplementary motor area; SMG, supramarginal gyrus; SPL, superior parietal lobule; STG, superior temporal gyrus; TP, temporal pole; VLPFC, ventrolateral prefrontal cortex.

**Table S3. The results of the meta-regression analyses showing effects of age and gender on disorder-gray matter volume (GMV) relationship ^a^**

| **Variable** | **Region** | **MNI coordinates** | **SDM-Z** | ***P* value** | **Cluster size** | **BA** |
| --- | --- | --- | --- | --- | --- | --- |
| **ADHD** | | | | | | |
| Mean age | R SFG | 22,60,8 | 1.918 | < .0001 | 112 | 10 |
|  | L SFG | -22,62,22 | 1.848 | < .0001 | 27 | 10/46 |
|  | R HPC | 22,-34,6 | -2.011 | .0001 | 23 | 27 |
| Female ratio | R HPC | 24,-32,-2 | 2.034 | .0002 | 14 | 37/27 |
|  | L MCC | 0,-16,40 | -2.420 | < .0001 | 156 | 23 |
| **SUDs** | | | | | | |
| Mean age | R SMG | 60,-26,18 | 2.853 | .0003 | 23 | 42/48 |
|  | L STG | -62,-26,6 | 2.870 | .0003 | 19 | 22/21 |
|  | L thalamus | 0,-20,4 | -1.725 | < .0001 | 139 | - |

^a^ Significant clusters were identified at *p* < .0005 and cluster size > 10 voxels.

Abbreviations: ADHD, attention-deficit/hyperactivity disorder; SUDs, substance use disorders; BA, Brodmann area; L, left; R, right; MNI, Montreal Neurological Institute; SFG, superior frontal gyrus; HPC, hippocampus; MCC, median cingulate gyrus; SMG, supramarginal gyrus; STG, superior temporal gyrus.

**Table S4. Jackknife sensitivity analyses of the studies included in the meta-analysis**

| Discarded study | ADHD | | | | Discarded study | SUDs |
| --- | --- | --- | --- | --- | --- | --- |
|  | L preCG | L SFG | R SFG | L IFG |  | L putamen |
| Bonath et al. 2018 | Y | Y | Y | Y | Aydin et al. 2009 | Y |
| Bralten et al. 2016 | N | Y | Y | N | Brooks et al. 2014 | Y |
| Brieber et al. 2007 | Y | Y | Y | Y | Bu et al. 2016 | Y |
| Depue et al. 2010 * | Y | Y | Y | Y | Cousijn et al. 2012 | Y |
| Iannaccone et al. 2015 | Y | Y | Y | Y | M. Dalwani et al. 2011 | Y |
| Kappel et al. 2015 | Y | Y | Y | Y | M. S. Dalwani et al. 2015 | Y |
| Lim et al. 2013 | Y | Y | Y | Y | Doallo et al. 2014 | Y |
| Ramesh et al. 2013 | Y | Y | Y | Y | Gilman et al. 2014 | Y |
| Saad et al. 2017 * | Y | Y | Y | Y | Hanlon et al. 2016 | Y |
| Stevens et al. 2012 | Y | Y | Y | Y | Howell et al. 2013 | Y |
| Van Dessel et al. 2020 | Y | Y | Y | Y | Koenders et al. 2016 * | Y |
| Vilgis et al. 2016 | Y | Y | N | Y | Mackey et al 2014 | N |
| Zhao et al. 2020 | Y | Y | Y | Y | Wang et al. 2014 | Y |
|  |  |  |  |  | Weiland et al. 2015 * | Y |
| Total | 12/13 | 13/13 | 12/13 | 12/13 |  | 13/14 |

*Note.* Y, the brain region remains significant in the voxel-based meta-analysis or sensitivity analysis compared to pooled findings; N, the brain region is no longer significant. * Studies reported null findings. Abbreviations: ADHD, attention-deficit/hyperactivity disorder; SUDs, substance use disorders; L = left; R = right; preCG, precentral gyrus; SFG, superior frontal gyrus; IFG, inferior frontal gyrus.

**References**

1. Frodl, T.,N. Skokauskas. Meta-analysis of structural MRI studies in children and adults with attention deficit hyperactivity disorder indicates treatment effects. *Acta Psychiatr Scand* 2012; 125(2): 114-26.

2. Lukito, S., L. Norman, C. Carlisi, J. Radua, H. Hart, E. Simonoff, et al. Comparative meta-analyses of brain structural and functional abnormalities during cognitive control in attention-deficit/hyperactivity disorder and autism spectrum disorder. *Psychol Med* 2020; 50(6): 894-919.

3. Pando-Naude, V., S. Toxto, S. Fernandez-Lozano, C.E. Parsons, S. Alcauter,E.A. Garza-Villarreal. Gray and white matter morphology in substance use disorders: a neuroimaging systematic review and meta-analysis. *Transl Psychiatry* 2021; 11(1): 1-18.
